# Supplementary material for: Characteristics and Application of a Novel Cold-Adapted and Salt-Tolerant Protease EK4-1 Produced by an Arctic Bacterium Mesonia algae K4-1
Source: Int J Mol Sci. 2023 Apr 28;24(9):7985. doi: 10.3390/ijms24097985 (PMC10178186; doi:10.3390/ijms24097985)
Supplement: Supplementary file 1 [file ijms-24-07985-s001.zip › ijms-2324516-supplementary.pdf]

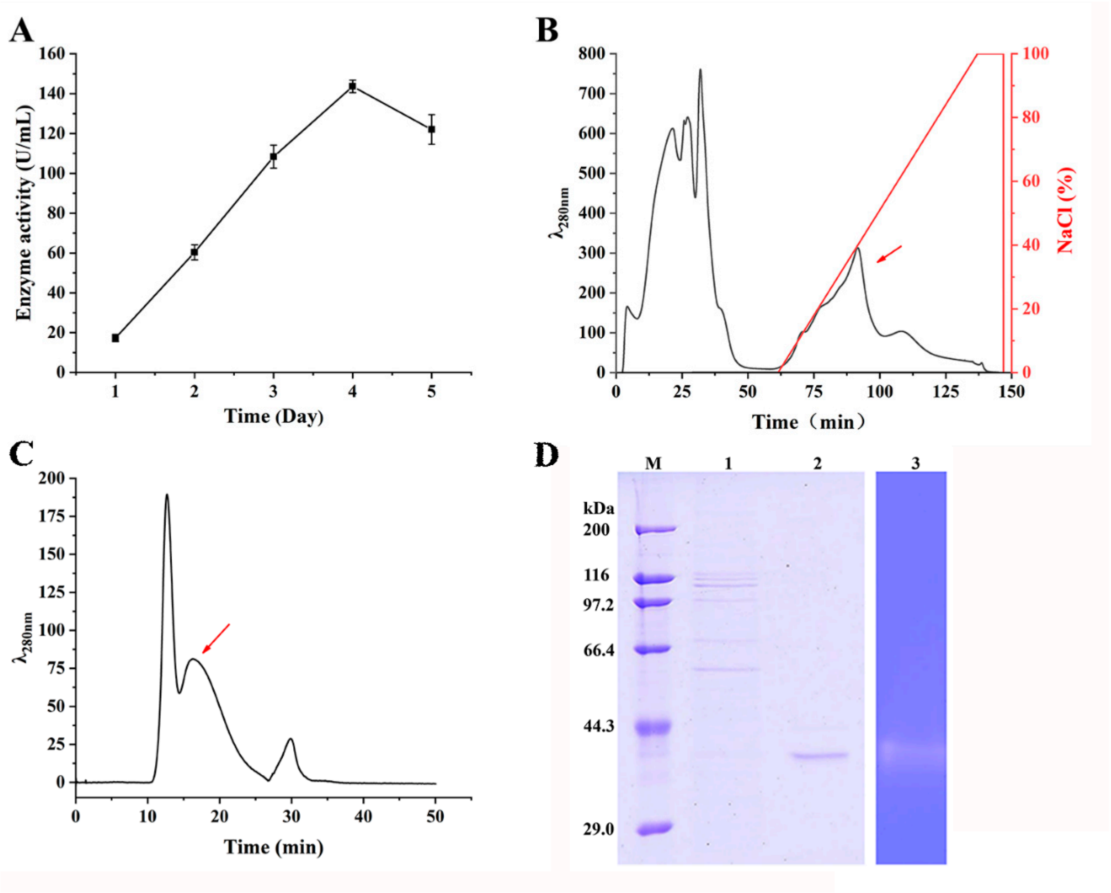

Figure S1. Separation and purification of protease EK4-1. (**A**): enzyme activity in fermentation broth; (**B**): anion exchange chromatography; (**C**): size-exclusion chromatography; (**D**): electrophoretic analysis of EK4-1. (1: fermentation crude enzyme solution, 2: denaturing band of molecular sieve sample, 3: active electrophoretic band of molecular sieve sample; Active components are shown by arrows in the figure)

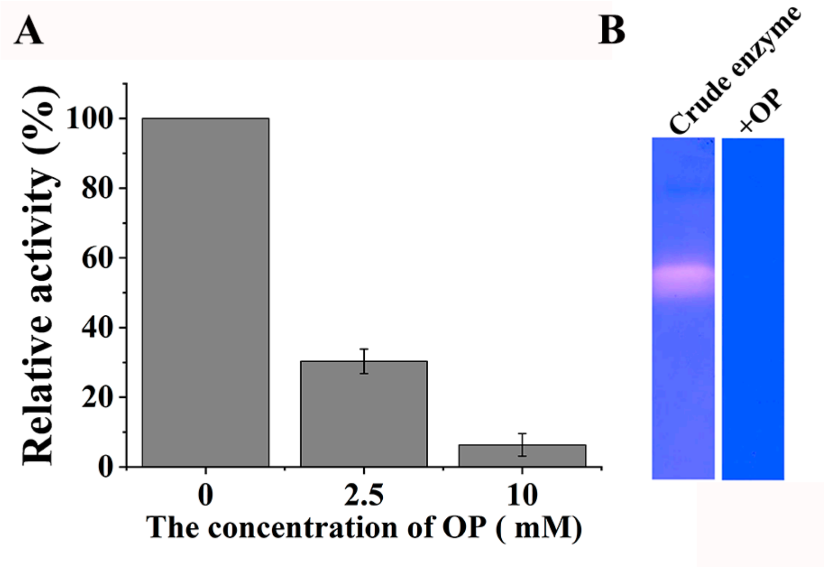

Figure S2. (**A**) Effect of metalloproteinase inhibitor OP on the activity of protease EK4-1 and (**B**) Zymogram results of inhibitor-casein immersion of protease EK4-1.
